# Choosing a sensible cut-off point: assessing the impact of uncertainty in a social network on the performance of NBDA

**DOI:** 10.1007/s10329-018-0693-4

**Published:** 2018-10-09

**Authors:** Sonja Wild, William Hoppitt

**Affiliations:** School of Biology, University of Leeds, Leeds, LS2 9JT UK

**Keywords:** Network-based diffusion analysis, NBDA, Social network, Uncertainty, Social learning

## Abstract

Network-based diffusion analysis (NBDA) has become a widely used tool to detect and quantify social learning in animal populations. NBDA infers social learning if the spread of a novel behavior follows the social network and hence relies on appropriate information on individuals’ network connections. Most studies on animal populations, however, lack a complete record of all associations, which creates uncertainty in the social network. To reduce this uncertainty, researchers often use a certain threshold of sightings for the inclusion of animals (which is often arbitrarily chosen), as observational error decreases with increasing numbers of observations. Dropping individuals with only few sightings, however, can lead to information loss in the network if connecting individuals are removed. Hence, there is a trade-off between including as many individuals as possible and having reliable data. We here provide a tool in R that assesses the sensitivity of NBDA to error in the social network given a certain threshold for the inclusion of individuals. It simulates a social learning process through a population and then tests the power of NBDA to reliably detect social learning after introducing observational error into the social network, which is repeated for different thresholds. Our tool can help researchers using NBDA to select a threshold, specific to their data set, that maximizes power to reliably quantify social learning in their study population.

**Electronic supplementary material:**

The online version of this article (10.1007/s10329-018-0693-4) contains supplementary material, which is available to authorized users.

## Introduction

Cultural behavior, broadly defined, is behavior that is passed on among individuals through social learning (Boyd and Richerson 1995). Therefore, if researchers are to understand the importance of cultural behavior in nonhuman primates and other animals, they need to be able to infer when social learning is responsible for the spread of behavior in natural settings. Recent years have seen the development of novel methods that quantify the importance of social learning on the spread of a behavior in freely interacting groups of animals. A method that has gained increasing popularity is network-based diffusion analysis (NBDA), a statistical tool that can quantify the effect of social learning among a group or population of animals (including humans) (Franz and Nunn 2009; Hoppitt et al. 2010). NBDA has been used in numerous studies to detect and quantify social learning in both free-ranging as well as captive animal populations across many taxa, including birds (e.g., Aplin et al. 2012; Boogert et al. 2014), insects (Alem et al. 2016), primates (Kendal et al. 2010; Schnoell and Fichtel 2012; Hobaiter et al. 2014) and cetaceans (Allen et al. 2013).

NBDA, first developed by Franz and Nunn (2009), infers social learning if the diffusion of a behavior follows the social network (i.e., a representation of connections among individuals within a social group or population), as it is based on the assumption that more closely associated individuals are also more likely to learn from each other (Coussi-Korbel and Fragaszy 1995). NBDA compares diffusion data with a matrix that contains a measure of association among individuals (Hoppitt et al. 2010), i.e., a measure of how frequently two individuals are observed together or in proximity. Diffusion data can either be the order with which individuals acquire a behavior (order of acquisition diffusion analysis–OADA) (Hoppitt et al. 2010) or it can be the time at which they acquire a behavior (time of acquisition diffusion analysis–TADA) (Franz and Nunn 2009; Hoppitt et al. 2010).

As both OADA and TADA track the spread of a novel behavior through the social network, accurate data on individuals’ network connections are desirable. Ideally, information on all individuals’ network connections is captured at once (Hoppitt and Farine 2017). However, for most studies on animal populations, especially free-ranging, this is not feasible, either due to sampling restrictions (time or space) or the inability to reliably identify all individuals, resulting in an incomplete record of all associations. Missing information can lead to imperfect relationships, which creates uncertainty about association strengths among individuals in the social network (Hoppitt and Farine 2017) with potential negative impacts on the power of NBDA to reliably quantify the importance of social learning (Hoppitt 2017).

Uncertainty decreases with the number of times an individual has been seen, and information on its connections with other individuals and estimates of association strengths between them gets more reliable. Several studies have outlined that collecting enough information on individuals’ associations is key to construct an accurate social network (Lusseau et al. 2008; Franks et al. 2010; Farine and Strandburg-Peshkin 2015; Silk et al. 2015). Thereby, the minimum number of observations for an accurate depiction of the social network depends on the level of social differentiation within the population, i.e., how varied the social system is, with more data required for populations with low social differentiation (Whitehead 2008). To minimize uncertainty, researchers often restrict their analysis by only including individuals above a certain threshold of sightings. A further argument for excluding animals with only few sightings when using NBDA is when not all individuals can be observed at all times and the target behavior is short or rare and hence easily missed by observers. In that case, a high cut-off point for the inclusion of animals can increase the certainty about an individual’s information status, i.e., to reliably distinguish if it is naïve or informed.

Franks et al. (2010) support the notion that sampling should in fact maximize the amount of data collected on known individuals, rather than maximizing the number of sampled individuals, as uncertainty in the social network is more problematic than missing individuals altogether. This is supported by Silk et al. (2015), who found that knowing even only 30% of individuals in a population can be enough to create informative social networks, as judged by network measures of connectivity at a node level. Both findings support having a large and conservative cut-off point for the inclusion of animals to reduce uncertainty in the network, if the aim is to make inferences about network structure.

However, it is less clear that a large, conservative cut-off point is appropriate when using NBDA. Dropping individuals with few sightings from a social network comes at the cost of information loss, if network connections between individuals are lost due to linking individuals being removed. For example, imagine that novel behavior is transmitted from A to B to C, where A and C are not directly linked. If B is removed due to a lack of data, it would appear that C has acquired the behavior by asocial learning and not by social learning. Even if the connections from A to B and B to C are inaccurately estimated, inclusion of B may nonetheless more accurately portray the transmission of information. Thus, missing network connections might result in lower power of NBDA to detect a social learning effect, and the recommendations of Franks et al. (2010) and Silk et al. (2015) may not stand for NBDA. Instead, having a lower threshold and including more individuals, while risking larger uncertainty in the network, may be preferable. Hence, there is a trade-off in the selection of a criterion for including individuals in the analysis between including as many as possible to have complete information on social network, and restricting inclusion of animals to reduce uncertainty (Bejder et al. 1998).

To resolve this issue, we provide a tool that can help researchers using NBDA to choose an appropriate threshold for the inclusion of individuals that maximizes the power of NBDA to reliably quantify social learning. For our simulations, we use OADA, which uses the order of acquisition as diffusion data. Our results still stand for TADA using continuous time data (Hoppitt et al. 2010); the log-likelihood function for continuous TADA is equivalent to the sum of the log-likelihood of the order of acquisition (used in OADA) and the log-likelihood for the time course of the diffusion independent of the identities of the learners. Thus, impacts of network inaccuracies on the power of OADA will similarly affect continuous TADA. Furthermore, results of the discrete time version of TADA (Franz and Nunn 2009) converge on those of the continuous TADA for small time periods (Hoppitt et al. 2010), suggesting that it will be similarly affected. Therefore, we suggest that researchers use the same technique described here to determine which individuals to include in a TADA, by omitting the time data from the procedure to determine the cut-off point that maximizes statistical power. Using a simulated data set, we simulate a learning process through the population and then assess the rate of false negatives (type 2 error) and false positives (type 1 error) of NBDA for different cut-off points after introducing noise into the social network. We furthermore assess if keeping individuals that learned (i.e., informed individuals) regardless of the number of times they have been seen, improves the power to detect social learning and assess the rates of false positives (type 1 error).

NBDA can also be applied to interaction data instead of association data (Franz and Nunn 2009; Hoppitt 2017). In this paper, we focus on the use of association networks since these have been most commonly utilized in NBDA—thus, the method we present is only directly applicable to association networks. However, the procedure could be modified to account for the sampling variation present in interaction data for differing observation periods across individuals.

## Methods

All simulations and analyses were run using R Studio v1.1.423 (R Core Team 2015). The supplementary material contains the R code to simulate observational data (OR1), the NBDA code (OR2) and the code for the simulations for assessing sensitivity of NBDA to observational error (OR3), the code for the application of the simulations to the simulated observational data (OR4), the simulated observational data (OR5) and resulting social network (OR6) and summary of results of all simulations (OR7–10), as well as a guide on how to use the codes (OR11) and further details on the algorithm with which the observational data was simulated (OR12) (Table 1).

**Table 1:** Structure of online resources

Online resource	File name	Content
OR1	Simulating data set	R code to simulate observational data for 60 individuals
OR2	NBDA code 1.2.15	R code NBDA
OR3	Sensitivity functions	R code for simulations on the sensitivity of NBDA to observational error for different cut-off points
OR4	Application to simulated data set	R code where we apply our simulations (OR3) the to the simulated observational data (OR5)
OR5	Simulated observational data	Csv file with simulated observational data
OR6	Social network	Csv file with association matrix resulting from simulated data set
OR7–OR10	Sensitivity summary	Csv files with summary of results of simulations applied to our simulated data set
OR11	How to use the code	Word document with guide on how to apply the sensitivity functions and specify the necessary parameters
OR12	Appendix	Word document that describes the algorithm we used to simulate observational data

### Input data set

In developing our methodology, we assume that researcher possess association data in an observation by individual matrix [see Farine (2013) for transformation of data], where a number of observations are made, with each individual in the population being recorded as being present during that observation (1) or absent (0). We assume these data are formatted as a matrix with observations (rows) × individuals (columns).

In order to test and illustrate the method developed, we simulated data of this form by developing an algorithm that resulted in a reasonable level of underlying social structure, which is necessary for NBDA to reliably detect social learning. We provide details of this algorithm in the supplementary material (OR12).

In our simulated data set, we obtained a group by individual matrix with 60 individuals and 331 observations (OR5). Group size varied from one to a maximum of ten individuals with a mean of 1.92 individuals per observation.

**Fig. 1:**
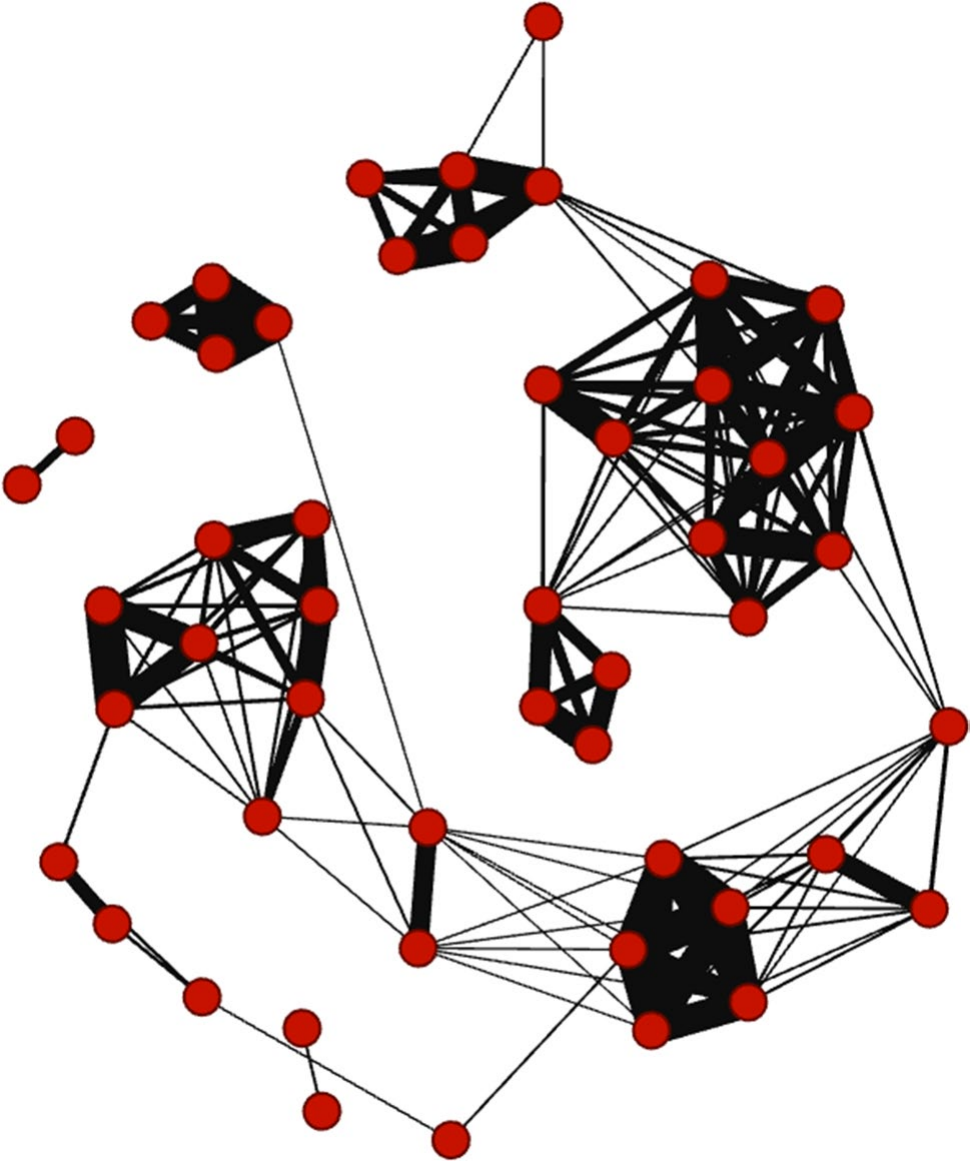
Weighted and undirected social network of a simulated data set with 60 individuals and 331 observations: Individuals (= nodes) are represented with* red circles*, associations between them (= edges) with* black lines*. The closer together nodes are and the thicker the edges, the stronger the association is between them

From the simulated data set, we created a social network using the simple ratio association index (for details see below) (R package ‘asnipe’) (OR6) for illustrative purposes (Cairns and Schwager 1987; Farine 2013; R Core Team 2015). Illustration of the social network (Fig. 1) was created using the Force Atlas 2 algorithm in Gephi (Bastian et al. 2009).

To this end, the algorithm we use to generate our illustrative data set arbitrarily assumes that 33% of individuals account for most of the observations in the association data. However, this is not an assumption of the procedure presented here, which accounts for the pattern of variability in the number of times individuals are observed in the specific data set being analyzed.

### Process overview

**Fig. 2:**
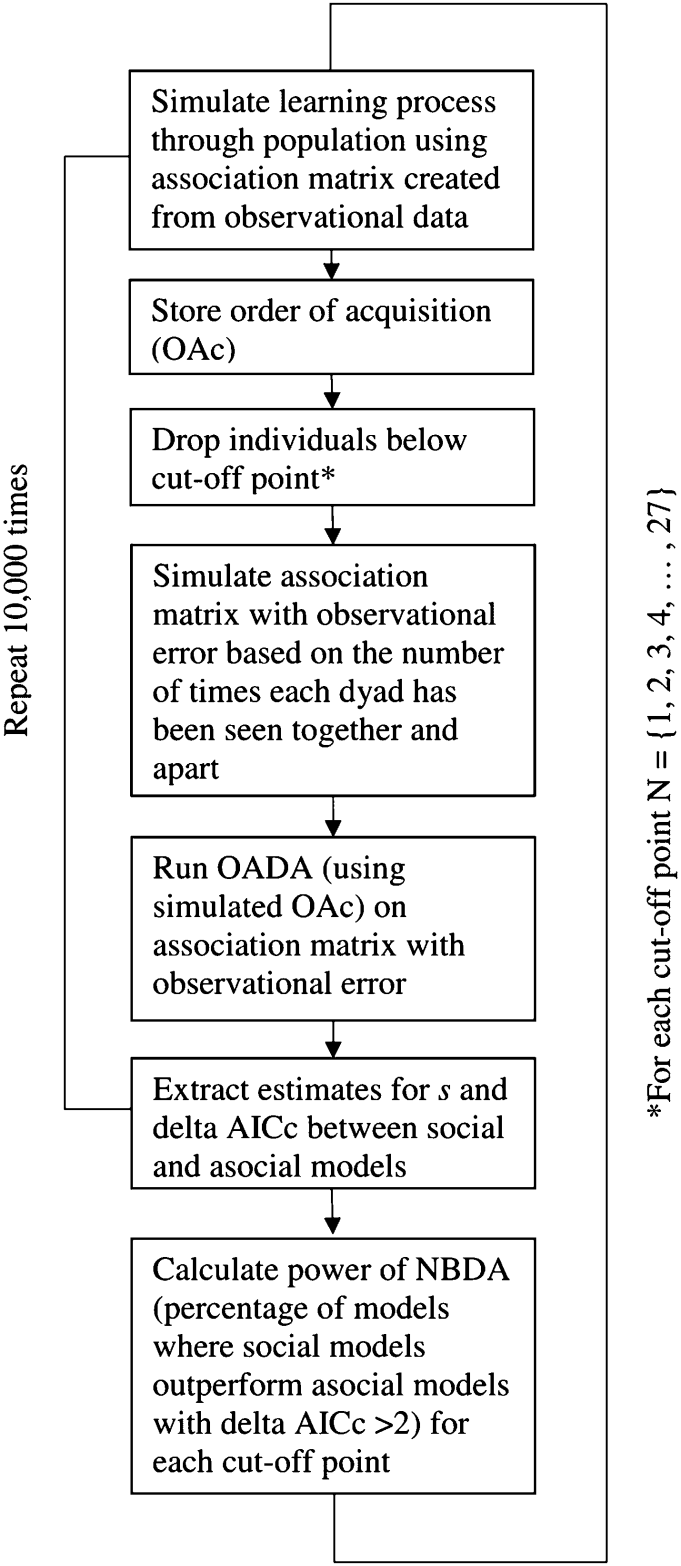
Flow diagram of simulation assessing the sensitivity of NBDA after introducing noise into the social network. *The user has an option to keep individuals who learned in the simulation, even though they would not make the cut-off

We developed a process that enables researchers to choose a justified cut-off point for the amount of association data (number of observations) that is required for inclusion of an individual into an NBDA (Fig. 2). This process consists of two steps: First, we simulated a social learning process, which we then analyzed using NBDA after introducing noise into the social network while applying different cut-off points for the inclusion of individuals to see which yielded the highest statistical power, i.e., the highest percentage of models where social learning correctly outperformed the null model with asocial learning. Secondly, we repeated the process of simulating a diffusion that was a result of only asocial learning to see which cut-off points yielded an appropriate false-positive error rate, i.e., where social models erroneously outperformed asocial learning models. We illustrate this process by applying both steps to the simulated data set described above.

To investigate if retaining learners (i.e., only excluding non-learners) influences power of NBDA to detect social learning, we then repeated the two steps, this time retaining all individuals who learned the behavior regardless of how many times they had been observed.

### Assessing statistical power of NBDA for different cut-off points after introducing observational error

Here, we propose a procedure for assessing the performance of NBDA, using different cut-off points, for a given data set. We first simulated a learning process through the population assuming learning follows the NBDA model. We then analyzed the resulting diffusion data using different cut-off points, and assessed the performance of NBDA to detect social learning in each case after introducing noise, i.e., observational error, into the social network.

As a first step, we created an association network from the simulated observational data. Association strengths (*a*_ab_) are usually estimated based on how many times two individuals (a and b) have been observed together as well as the number of times they have been seen apart [for guidance on choosing an appropriate association index, see Cairns and Schwager (1987); Hoppitt and Farine (2017)]. We used the ‘simple ratio association index’ (hereafter “SRI”) (Cairns and Schwager 1987), which is defined as

$${\text{SRI}} = \frac{x}{{y_{\text{a}} + y_{\text{b}} + y_{\text{ab}} + x}} ,$$

where *x* is the number of sampling periods individuals a and b were observed associated, *y*_a_ is the number of sampling periods with just a identified, *y*_b_ is the number of sampling periods with just individual b identified, and *y*_ab_ is the number of sampling periods where both individuals a and b were identified, but not in association.

As a next step, we modeled a diffusion (the documented spread of a novel behavior pattern) using the resulting social network from the simulated data (*N* = 60, 331 observations): In a first round, one individual was randomly chosen to learn. In each subsequent round, we calculated the total association with informed individuals for each individual. Following the NBDA model (Hoppitt et al. 2010) we then calculated an individual’s learning rate *R*_i_ as

$$R_{i} = s \times \sum\limits_{j = 1}^{n} {a_{ij} + 1} ,$$

where *s* denotes the social learning parameter, which estimates the strength of social learning per unit association with informed individuals relative to the average rate of asocial learning; and $\sum\nolimits_{j = 1}^{n} {a_{ij} }$ the total association of individual *j* with informed individuals. Here, *s* represents the strength of social learning relative to asocial learning and must be set by the user. The ultimate aim of this process is to determine which cut-off point has most power to detect social learning. If *s* is set too high, then all simulations will have high power, whereas if *s* is set too low, all simulations will have low power. The user must find a value of *s* (by trial and error) that results in a range of statistical power, in order to determine which cut-off point is most likely to detect social learning if it is occurring. For this simulation, we set *s *= 8, which corresponds to an eight-fold increase of the social learning rate per unit association with informed individuals compared to an individual’s asocial learning rate. The probability that each individual was next to learn is then given as:

$$\frac{{R_{i} }}{{\sum\nolimits_{j} {R_{j} } }}$$

This process was repeated until 20 individuals had acquired the behavior (this represents an arbitrarily chosen number of learners—in practice this would be matched to the actual number observed to learn in the population).

As a third step, we used a Bayesian approach to simulate a social network that introduced a level of error for each dyad that depended on the number of times each dyad had been seen together and the number of times they had been seen apart. Thereby, the more often members of a dyad had been seen, the closer their simulated association strength was to the real value. Similarly, if a dyad had only been seen a handful of times, the simulated values would be more varying (more noise) and potentially further away from the real value. Since the value of *a*_*ij*_ is a proportion (proportion of times *i* and *j* are expected to be seen together), knowledge about *a*_*ij*_, given the data available, can be modeled as a beta distribution (known as the ‘conjugate’ prior distribution for a proportion) with parameters *a* and *b*:

$$a_{ij} \sim {\text{Beta}}(a,b) .$$

When we have no data, we set *a *= *b *= 1, which gives a uniform distribution for *a*_*ij*_—i.e., we accept that *a*_*ij*_ is equally likely to take any value from 0 to 1. After we collect data, we update our prior distribution for *a*_*ij*_ to yield a posterior distribution, giving our updated knowledge about *a*_*ij*_.

After collecting data of *n* independent observations, the posterior distribution for *a*_*ij*_, given *x*, is given by

$$a_{ij} \left| {{\text{data}} \sim {\text{Beta}}(a + x,b + n - x)} \right.$$

where *x* represents the number of successes (i.e., number of times two individuals have been seen together) and *n* − *x* the number of failures (i.e., number of times two individuals have been seen apart). The smaller *n* is, the wider the beta distribution will be, reflecting our increased uncertainty in the value of *a*_*ij*_. This conjugate method of updating our knowledge about a proportion based on independent Bernoulli trials is a standard and accepted method in Bayesian statistics. Therefore, this method of calculating the level of uncertainty is appropriate for the common situation where association data is used to calculate the SRI with observations sufficiently spaced out that they can be considered independent. Researchers could, in principle, substitute an alternative appropriate expression for error for other indices (e.g., Hoppitt and Farine 2017).

Hence, from the sightings record, we created a matrix containing the number of times each dyad had been observed together (successes). A second matrix contained the cumulative number of times each member of a dyad had been observed without the other individual in the dyad (failures). We provide a function that extracts said matrices from the observation record (OR3). Making no assumptions about the distribution of the association strengths within the social network, we used an uninformative (uniform) prior beta(1,1). We then simulated association strengths *a*_*ij*_ using

$$a_{ij} \left| {{\text{data}} \sim {\text{Beta}}(1 + x,1 + n - x)} \right.$$

The resulting association matrix represented a social network with noise and was used to test for statistical power of NBDA for different cut-off points. For our simulated data, we used cut-off points of *N* = {1, 2, 3, 4, 5, 6, 7, 8, 10, 11, 13, 14, 15, 16, 17, 18, 19, 21, 22, 24, 26, 27}. In each case, all individuals with fewer sightings than *N* were dropped from the social network. Note that the user has the option to keep individuals who learned and only drop non-learners (as described at the bottom of the Methods section).

The fourth step was to test the performance of NBDA to correctly identify social learning. We ran the OADA (‘order of acquisition diffusion analysis’ (Hoppitt et al. 2010) variant using the simple ratio association matrix (simulated—with observational error) and the simulated order of acquisition that was obtained using the error-free network.

From the OADA model, we extracted the estimates for the social learning parameter *s*, the *p* value of the likelihood ratio test, the AICc (Akaike information criterion corrected for small sample size) (Burnham and Anderson 2002) values for the social models as well as the models where *s* was constrained to zero, i.e., the asocial models. To select a model over an alternative model, differences in AICc values (delta AICc) need to cross a certain threshold (defaulted to 2 in our simulation; can be set by user) (Burnham and Anderson 2002). We hence calculated the delta AICc value between social and asocial models. We furthermore recorded if the true value of *s* was within the 95% confidence interval (CI) for set *s* (i.e., within 1.92 units) and if outside of the confidence interval, we determined if *s* was an under- or overestimate. The whole process was repeated 10,000 times for each cut-off point.

As a last step, we calculated the percentage of models where the delta AICc value was above the set threshold of 2, i.e., where one model (social or asocial) was outperforming the alternative model. From those models, we calculated (1) the mean and standard deviation for the estimates of *s* for each cut-off point; (2) the percentage of models where social models performed better than asocial models—giving the power of NBDA to detect social learning; (3) the percentage of models where the true value of *s* fell within the 95% CI for *s* (this should be ~ 95% if the model is performing well); and (4) the percentage of models that over- or underestimated the value of *s*, i.e., were above the upper limit of the 95% CI or below the lower limit, respectively (this should be approximately even if NBDA is performing well).

We then repeated the entire process, but this time retaining all individuals who had learned, irrespective of how many times they had been sighted, i.e., only excluding individuals that did not learn. We provide the option to retain all learners in our code.

## Assessing the false positive error rate in NBDA for different cut-off points

In order to assess the rate of false positives, i.e., where NBDA identifies a social learning effect where there is in fact none, we repeated the procedure described above, but this time constraining *s *= 0, which corresponds to learning asocially, i.e., through independent innovations. We assessed the rate of false positives for both models where individuals were dropped regardless of their information status as well as models where learners were retained regardless of how many times they had been sighted.

## Results

### Assessing statistical power of NBDA for different cut-off points

In an NBDA, social learning is inferred if the AICc for the model including social learning is lower than for a model without social learning. Therefore, the percentage of occasions that social learning models outcompete asocial learning models gives a measure of statistical power for each cut-off point, when *s *> 0.

**Fig. 3:**
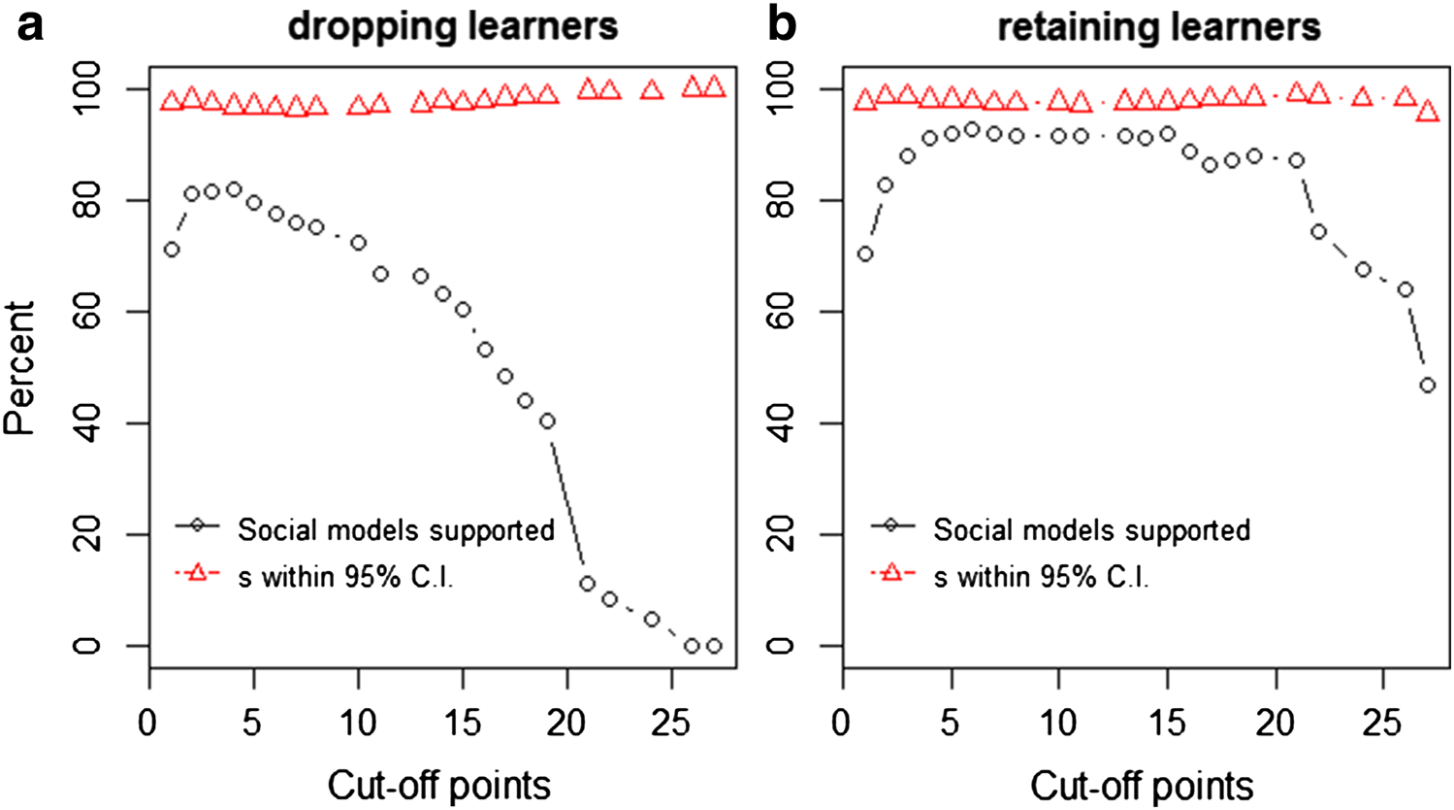
Power of NBDA to correctly identify social learning after introducing noise into a social network (*black circles*) and percentage of models where estimates for the social learning parameter *s* fell within the 95% CI of the set *s* (= 8) for a given cut-off point (*red triangles*) for **a** models where all individuals were dropped below the cut-off point and **b** models where learners were retained regardless of how many times they had been observed

For models where all individuals were dropped with sightings below the cut-off point (regardless of information status), statistical power was highest at a cut-off point of 4 with 83.96% power (Fig. 3a, OR7). Averaged estimates for *s* were consistently higher than set in the simulation (*s *= 8), ranging from 307.68 to 784.11 (OR7). Estimates for *s* followed an upwards trend as the cut-off point increased (OR7)—we explain why this occurs in the Discussion section below. In 96.51%–100% of models—depending on the cut-off point—the true value of *s* (8) fell within the 95% CI (Fig. 3a, OR7), suggesting that the 95% CI for *s* can be trusted as being appropriate for all cut-off points, if *s *> 0.

For models where learners were kept regardless of how many times they were observed, power to correctly detect social learning was highest at cut-off point 6 with 92.95% power (Fig. 3b, OR8). Averaged estimates for *s* ranged between 267.23 and 383.64, and in between 95.59 and 99.19% of models—depending on the cut-off point—the true value of *s* (8) fell within the 95% CI (Fig. 3b, OR8). For all cut-off points, retaining learners increased the power to detect social learning compared to when learners were dropped.

### Assessing the false-positives error rate in NBDA for different cut-off points

**Fig. 4:**
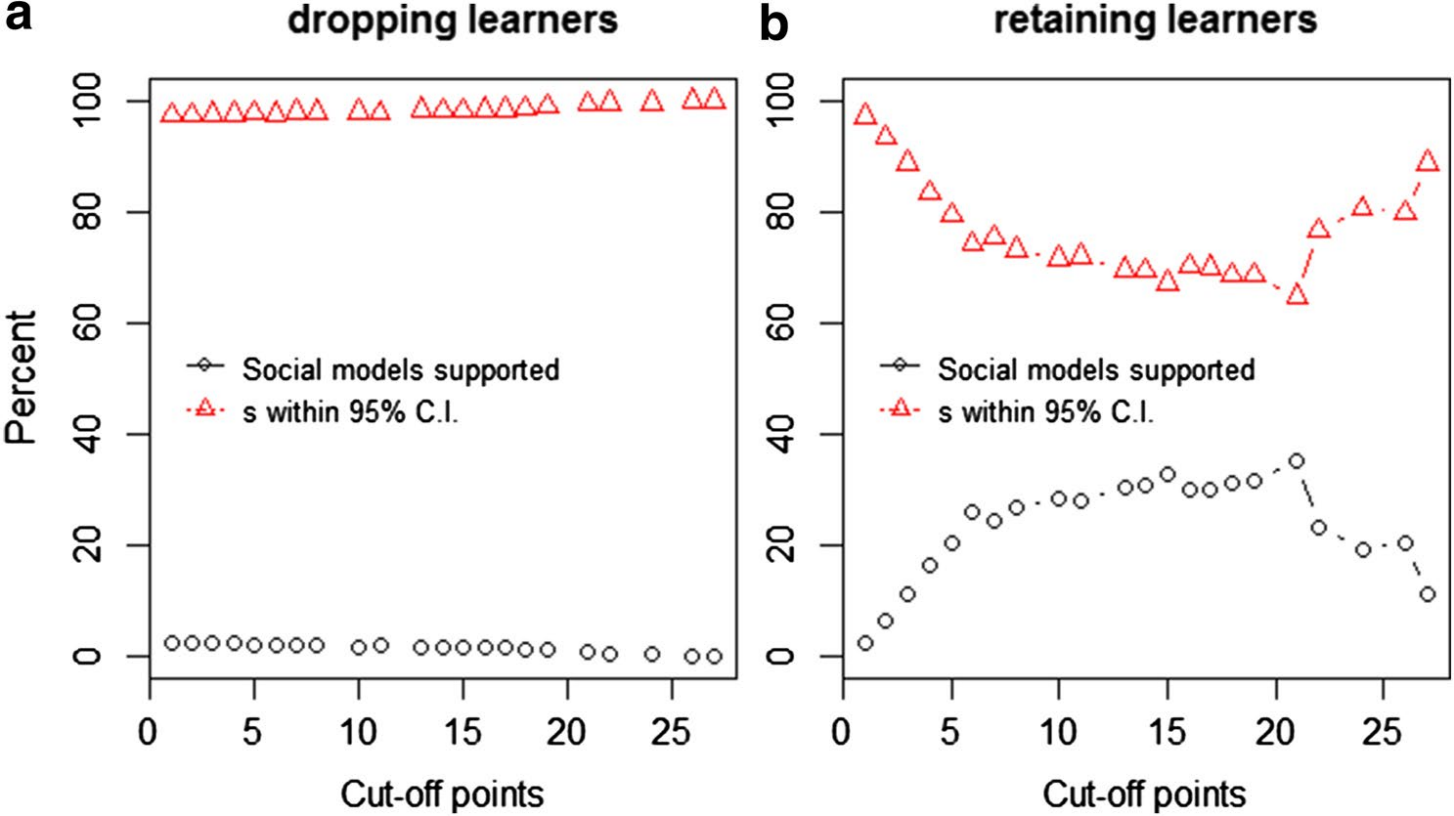
Percentage of models where NBDA incorrectly identifies social learning after introducing noise into a social network (*black circles*) and percentage of models where estimates for the social learning parameter *s* fell within the 95% CI of the set *s* (= 0) for a given cut-off point (*red triangles*) for **a** models where all individuals were dropped below the cut-off point and **b** models where learners were retained regardless of how many times they had been observed

For models where all individuals were dropped below the cut-off regardless of their information status, the percentage of models where social learning was incorrectly outperforming asocial models when *s *= 0 (false positives) ranged between 0 and 2.23% (Fig. 4a, OR9). Therefore, for these data, the false-positive error rate was always below that commonly accepted (5%) (‘commonly’ refers to all statistics that consider a *p* value of < 0.05 as statistically significant). In this case, a researcher could safely choose whichever cut-off point gave the highest statistical power. Averaged estimates for *s* were again consistently higher than set in the simulation (*s* = 0), ranging from 23.18 to 488.5 and exponentially increasing with an increasing cut-off point (Fig. 4a, OR9). The true value of *s* (0) fell within the 95% CI of *s* in 97.37–100% of the models depending on the cut-off point (Fig. 4a, OR9), further supporting the fact that, for this data, all cut-off points can be trusted.

For models where informed individuals (learners) were retained, the percentage of models where social models incorrectly outperformed asocial models (for *s *= 0) ranged between 2.37 and 35.14%, and was for most cut-off points above the commonly accepted 5% (Fig. 4b, OR10). Averaged estimates for *s* ranged between 38.23 and 245.28 and the true value of s (= 0) fell within the 95% CI of the estimated *s* in 64.83–97.2% of models (Fig. 4b, OR10).

## Discussion

We present a method for choosing a cut-off point for the inclusion of individuals in an NBDA based on the number of times they are observed in the construction of the social (association) network. Above, we illustrated this process by applying it to a simulated data set. Below we discuss how the results obtained could be used to select an appropriate cut-off point for this data set. There is no reason to think that the cut-off point identified for our simulated data would be applicable in general—the appropriate cut-off point will depend on the properties of the data set in question. Nonetheless, the same logic could be used to choose a cut-off point for real data sets.

After simulating a learning process through a social network, we used a Bayesian approach to simulate a social network that introduced a level of error for each dyad depending on the number of times each dyad had been seen together and apart. The diffusion data and the social network with observational error were then analyzed using NBDA to find an appropriate cut-off point for the data set.

The same approach could be used to estimate the impacts of noise in a social network for real NBDA data, where the social network is constructed from association data. We provide a function that allows the extraction of one matrix with the number of times each dyad has been seen together and a second matrix with the number of times they have been seen apart, which can then be used to simulate an association network including noise. Alternatively, users can provide their own matrices for the simulation. The approach presented here is for an NBDA that assumes a static association network that is essentially unchanging over time. NBDA itself can be extended to allow the use of a dynamic instead of a static network (Hobaiter et al. 2014), but further work is needed to determine cut-off points under such circumstances.

The simulations presented then allow researchers to test (1) the statistical power and (2) the false-positive error rate of NDBA under different cut-off points. In our simulated data set—for models where all individuals were dropped below the cut-off point regardless of their information status—false positive error rates were appropriate across the range of cut-off points. Furthermore, for both *s *= 0 and *s *= 8, the true value of *s* was within the 95% CI approximately 95% of the time. This suggests that the validity of NBDA could be trusted for any cut-off point, and so the cut-off point should be chosen to maximize statistical power. Our results correspond broadly with Hoppitt’s (2017) finding that error in the network does not increase false positives in NBDA but can act to make the analysis more conservative in detecting social learning (see also Whalen and Hoppitt 2016). However, it is uncertain if invariability of false error rate to cut-off point choice is a general feature of NBDA, so we encourage researchers to always check their own data set before accepting a cut-off point.

In our simulated data set, power of OADA was maximized at a cut-off point of four sightings, which would result in the inclusion of 41 out of the 60 individuals (= 68%). Silk et al.’s (2015) finding that having data on as little as 30% of the population allows to create an informative social network, does not prove to be true for NBDA using our simulated data, as power to detect social learning dropped to only 60% with 20 individuals being included (which corresponds to a 30% threshold). Thus, we show that Silk et al.’s (2015) threshold is not generally appropriate for NBDA (and was not suggested for this purpose). Instead, the threshold where power of OADA is maximized is likely to vary depending on the specific data set—the number of individuals in the population, the length of diffusion, association strengths among individuals and the social differentiation of the population, i.e., how varied the social system is (Franz and Nunn 2009; Hoppitt et al. 2010). Hence, we recommend using our proposed methods to ensure a threshold for the inclusion of animals that is specific to the data set, and discourage the use of arbitrarily chosen thresholds when using NBDA.

For models where all informed individuals were kept regardless of how many times they had been seen, power of NBDA to detect social learning was highest at a cut-off point of 6. Furthermore, power to detect social learning was higher compared to when learners were dropped. Dropping individuals will intuitively reduce power to detect social learning when linking individuals are being removed (as explained in the Introduction). However, false-positive rates (when s = 0) for most cut-off points was high when all learners were retained (and above the commonly accepted 5%) for all but one cut-off and over 25% for the cut-off point of 6 which yielded highest statistical power. Hence, even though keeping learners may improve the statistical power to detect social learning, it may also substantially increase the risk of false-positive results. Therefore, the option to keep all informed individuals in the simulation should only be made use of after ensuring that the rate of false positives falls below the 5% threshold for the chosen cut-off point. For our simulated data set, we would conclude that we should drop learners if they do not make the cut-off point, since risking a 25% chance of a false-positive result would make a positive result untrustworthy. We suspect that it may prove to be a general pattern that retaining all learners results in an unacceptable false-positive error rate.

In order to run the simulation to assess statistical power, researchers must choose a value of *s*. In a sense, this choice is arbitrary, since, allowing for sampling error, power will peak at approximately the same point for all *s *> 0. However, if *s* is set high, then statistical power will appear level at 100%, if *s* is set too low, statistical power will appear level at 0%. Therefore, some trial and error may be required to find a useful value for *s*.

In all simulations on the sensitivity of NBDA (with *s *= 8 and *s *= 0, both with dropping and keeping learners), average estimates for the social learning parameter *s* across simulations were considerably higher than the true values set, even though when learners were dropped. Nevertheless, in ~ 95% of cases the true value of *s* fell within the 95% CI as would be expected if OADA was performing well. Hoppitt (2017) found the same effect in OADA. The bias arises because in cases where the diffusion follows the network very closely, the likelihood of the data increases and plateaus as *s* increases to infinity. Thus the optimization algorithm used to fit the model converges on an arbitrarily large value for the estimate of *s*, which biases the average value of estimates of *s* upwards. In such cases, there is also no upper limit for the 95% CI of *s*. Therefore, this is not a reason to generally distrust estimates of *s* obtained from an OADA. Instead, one should mistrust the estimated value of *s* if it appears unrealistically high and there is no upper limit for its 95% CI. In such cases one can still take the lower bound of the 95% CI as providing a lower plausible limit on the strength of learning. The upper bound of infinity is merely indicating that it is plausible that everyone in the population who learned the behavior while connected to an informed individual, did so by social learning. Overall, the results obtained here and by Hoppitt (2017) indicate the best way to interpret OADA is to consider the 95% CI as a plausible range of values for *s*, as opposed to focusing on the value of the maximum likelihood estimate for *s*. Given this is a sensible strategy for interpreting the outputs of any statistical model, this is unlikely to represent a severe limitation of OADA.

NBDA has gained increasing popularity to detect social learning in both captive and free-living populations of various species. It has proven to be a useful tool to detect and quantify social learning in animal (and human) populations (e.g., Kendal et al. 2010; Hoppitt et al. 2010; Aplin et al. 2012; Allen et al. 2013; Alem et al. 2016). We show that previously proposed thresholds for the inclusion of animals for building networks may not be applicable to studies using NBDA. Hence, we strongly encourage researchers to use our simulation to choose a cutoff point that maximizes power of NBDA that is specific to their data set, and discourage the use of arbitrarily chosen thresholds in order to minimize the risk of false negative and positive results.

## Electronic supplementary material

Below is the link to the electronic supplementary material.

Supplementary material 1 (.r 7 kb)

Supplementary material 2 (.r 15 kb)

Supplementary material 3 (.r 153 kb)

Supplementary material 4 (.r 10 kb)

Supplementary material 5 (CSV 42 kb)

Supplementary material 6 (CSV 12 kb)

Supplementary material 7 (CSV 2 kb)

Supplementary material 8 (CSV 2 kb)

Supplementary material 9 (CSV 2 kb)

Supplementary material 10 (CSV 2 kb)

Supplementary material 11 (DOCX 45 kb)

Supplementary material 12 (DOCX 16 kb)
