# Supplementary material for: Choosing a sensible cut-off point: assessing the impact of uncertainty in a social network on the performance of NBDA
Source: Primates. 2018 Oct 9;60(3):307–15. doi: 10.1007/s10329-018-0693-4 (PMC6459781; doi:10.1007/s10329-018-0693-4)
Supplement: Supplementary file 11 — Supplementary material 11 (DOCX 45 kb) [file 10329_2018_693_MOESM11_ESM.docx]

‘NBDA – SENSITIVITY USER GUIDE V 1.0’

WRITTEN BY SONJA WILD & WILLIAM HOPPITT

Our code provides a tool in R to infer sensitivity of network-based diffusion analysis (NBDA) to uncertainty in social networks: the function **together_apart** creates an array with two matrices with measures of how many times each dyad has been seen together and how many times they have been seen apart. The function **sensitivity_NBDA_ind_error** then assesses the power of NBDA to reliably quantify social learning after introducing uncertainty into the social network (using the together/apart matrices) and can help researchers to find an appropriate threshold for the inclusion of individuals.

Last update: 13.06.2018

Contents

[1. Dependent packages and NBDA code 3](#_Toc508096291)

[2. Input Data 3](#_Toc508096292)

[3. Extracting together/apart matrices 3](#_Toc508096293)

[3.1. Together_apart function and arguments 4](#_Toc508096294)

[3.2. Examples 4](#_Toc508096295)

[3.3. Output 4](#_Toc508096296)

[4. Sensitivity of NBDA using different cut-off points and noise in social network: false negatives and false positives 4](#_Toc508096297)

[4.1. Sensitivity_NBDA_ind_error function and arguments 4](#_Toc508096298)

[4.2. Examples 5](#_Toc508096299)

[4.3. Output 5](#_Toc508096300)

[5. Computational power and number of iterations 5](#_Toc508096301)

[References 6](#_Toc508096302)

# Dependent packages and NBDA code

Install packages: *‘asnipe’* (Farine 2013) *‘survival’* (Therneau and Lumley 2017) *‘Matrix’* (Bates and Maechler 2007) *‘matrixStats’* (Bengtsson et al. 2014) *‘combinat’* (Chasalow 2012) *‘foreach’* (Calaway et al. 2015) *‘doParallel’* (Calaway et al. 2015) *‘tcltk’* (R Core Team)

All codes (generating together/apart matrices, sensitivity function and application to simulated data set) are available in the supplementary material.

The most up to date version of NBDA (Hoppitt) is available on <https://lalandlab.st-andrews.ac.uk/freeware/>

**IMPORTANT:** The sensitivity code has to be able to source the NBDA code due to parallel processing. The simulations will therefore only work if the working directory has been set to the folder containing the NBDA code and the NBDA code must be labelled “NBDA code 1.2.15.R”.

# Input Data

The input file needs to be a group by individual matrix (Figure 1). Each row represents a sighting or group, each column represents an individual. Each individual gets a binary entry of 1 if present in that group or 0 if absent. Data in many other formats (e.g. from linear mode) can be transformed using the R package ‘*asnipe*’ (Farine 2013).


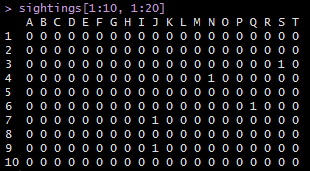


Figure 1: A group by individual matrix is needed as an input file.

# Extracting together/apart matrices

The certainty about the levels of associations within an individual’s social network increases with the number of times an individual has been seen. In order to test the impact of noise in the social network, we simulate an association matrix based on how many times each dyad has been observed together and how many times they have been observed apart. The function **together_apart** extracts those numbers from the sightings record and stores the two matrices in an array. These are then used as a basis to simulate an association matrix for the simulation part, representing a social network with noise. Alternatively to using our provided function, the user can choose to provide their own array with their own matrices of a measure of ‘together’ and ‘apart’ if so wished.

## Together_apart function and arguments

**Function:**
together_apart (sightings)

**Parameters:**

sightings: group by individual matrix with binary entries (1,0) (see section 2)

## Examples


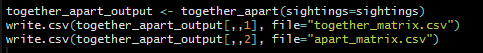


## Output

The output is an array with two matrices, the first one containing the number of times each dyad has been seen together and the second one containing the number of times members of a dyad have been seen apart. Ensure to save the output to an object, which can then be used for the simulation on the sensitivity of NBDA.

# Sensitivity of NBDA using different cut-off points and noise in social network: false negatives and false positives

This function simulates a learning process through the population and stores the simulated order of acquisition. As a second step, individuals below a certain threshold are dropped. Here, the user has the option to either drop all individuals below the threshold (regardless of their information status) or can choose to retain all individuals that learned (regardless of how many times they have been seen). The third step is to run OADA using the order of acquisition on a simulated association network (using the together/apart matrices to generate them). When s>0, one can estimate the power of NBDA to detect social learning for each cut-off point. When s = 0 one can obtain the rate of false positives (type 1 error) for each cut-off point.

## Sensitivity_NBDA_ind_error function and arguments

**Function:**sensitivity_NBDA_ind_error (x, sightings, cutoff, association_index, iterations, s, num_ind_learn, cores, keep_learners, delta_AICc)

**Parameters:**

x: name of the object from the together_apart function (array with two matrices) (see section 3.3)

sightings: group by individual matrix with binary entries (1,0) (see section 2)

cutoff: a vector with the values of the desired thresholds for the inclusion of individuals (number of sightings). The cut-off point can be as low as 1, while the upper limit has to be chosen so that at least 2 individuals make the cut-off point.

association_index: either “SRI” for simple ratio association index or “HWI” for half-weight association index (calculations are based on *get_network* function of package *asnipe*.

iterations how many times the learning process is simulated and OADA is run on the simulated order of acquisition.

s social learning parameter s. If set to 0, one can test for the rate of false positive results (type 1 error). If set to >0, one can test for the power of NBDA.

num_ind_learn Number of individuals in the population that learn the behaviour. This should be matched with the real number of individuals that learned.

cores Number of cores the simulations run on in parallel. If unspecified, simulations are set to run on all available cores.

keep_learners If FALSE, all individuals are dropped below the specified threshold. If TRUE, all learners are kept regardless of how many times they have been seen.

delta_AICc minimum difference in AICc value to select one model (either social or asocial) over the alternative. Defaulted to 2.

## Examples


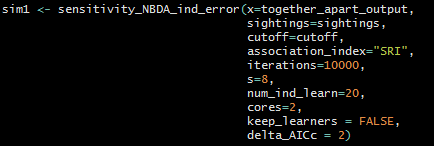


## Output

An object with two slots:

$raw Raw data table with each row representing one model: Columns specify the cut-off point, the number of individuals included, the number of individuals that acquired the behaviour, the iteration of the respective model, AICc values for both social (aicc) and asocial models (aiccNull), delta AICc, p values, estimates of the log likelihood of the estimated s and set s, a column specifying if the log likelihood of the estimated s falls within the 95% confidence interval of the set s (yes/no) and a column specifying if s is an under- or overestimate (under/over) given the log likelihood falls outside the 95% confidence interval.

$summary A summary table with models averaged for each cut-off point. Columns specify the cut-off point, the number of individuals included, the number of individuals that acquire the behaviour, delta AICc, percentage of models where one model outperforms the other (delta AICc above set threshold), the percentage of models where social learning outperforms asocial learning, the set s, averaged estimates and standard deviations for s, proportion of models where the log likelihood of s falls within the 95% confidence interval of the set s, a column specifying in what percentage of the cases s is an overestimate or an underestimate respectively (given the log likelihood falls outside the 95% confidence interval).

# Computational power and number of iterations

Extracting the together/apart matrices can be computationally intense for very large data sets (hundreds of individuals) and may take up to several hours. Furthermore, the simulation (sensitivity of NBDA) is set up to run in parallel on multiple cores if required, as - depending on the size of the data set - the simulation requires a lot of computational power. To ensure robustness of the results, >1000 iterations as a minimum are recommended, but robustness increases with the number of iterations.

# References

Bates D, Maechler M (2007) Matrix: A Matrix package for R. R Packag version 099875-2, URL http//CRAN R-project org 15:

Bengtsson H, Corrada-Bravo H, Gentleman R, Jaffee H (2014) matrixStats: Methods that apply to rows and columns of a matrix. R Packag version 08 14:

Calaway R, Weston S, Tenenbaum D, Revolution Analytics (2015) doParallel: Foreach parallel adaptor for the “parallel”package. R Packag version 1:

Chasalow S (2012) Combinat: combinatorics utilities. R package version 0. 0-8

Farine DR (2013) Animal social network inference and permutations for ecologists in R using asnipe. Methods Ecol Evol 4:1187–1194 . doi: 10.1111/2041-210X.12121

Hoppitt WJE Network Based Diffusion Analysis (NBDA) Version 1.2. http://lalandlab.st-andrews.ac.uk/freeware/

R Core Team Package “tcltk”

Therneau TM, Lumley T (2017) Package “survival”
