# Supplementary material for: Choosing a sensible cut-off point: assessing the impact of uncertainty in a social network on the performance of NBDA
Source: Primates. 2018 Oct 9;60(3):307–15. doi: 10.1007/s10329-018-0693-4 (PMC6459781; doi:10.1007/s10329-018-0693-4)
Supplement: Supplementary file 12 — Supplementary material 12 (DOCX 16 kb) [file 10329_2018_693_MOESM12_ESM.docx]

**Generation of simulated association data**

We first simulated a data set with 60 animals. The goal here was simply to generate a typical set of data to test the proposed method developed. The form of the data is similar to the data typically collected by researchers aiming to construct social networks based on association data (for transformation of data formats see Farine (2013)). We generated a binary data matrix: where each row represents an observation or group, whereas each column represents an individual, with 1 denoting presence in that observation and 0 denoting absence. To generate data of this form, we first simulated how many times each individual was seen using Poisson distributions with 5 different means (25 individuals with a mean of 1, 15 individuals with a mean of 5, 10 individuals with a mean of 20 and 5 individuals each with a mean of 20 and 30 respectively), in random order. This was done as a method to represent heterogeneity in the sampling observations/detections of individuals.

We first simulated the presence/absence of the first individual A in 600 observations by randomly attributing a 1 (presence) to the observations with a probability of n/600 (n = number of sightings for individual A) and 0 (absence) otherwise. For the next, randomly chosen, individual (e.g. individual B), we first chose an associate (i.e. an animal whose sightings had already been assigned), which in the first round was only individual A. We then determined if the two individuals were associated (with a probability of 0.7 of them being associated) and if so, we simulated the strength of association using a uniform distribution ranging between 0.5 and 1. For each observation in which the A was present, we simulated B’s presence using their association strength as a probability of being present. For observations in which A was absent, we first extracted B’s simulated number of sightings (from the Poisson distribution) and subtracted both the number of times it had already been seen without A as well as the expected number of times it would be seen together with A (based on their probability of association). This resulted in an estimate of the remaining number of sightings where we expected B to be seen without A. We divided this number by the remaining number of sightings where no other individual was present to receive a probability of B’s presence in observations where A was absent. To reduce noise, we multiplied the probability of B’s presence – in absence of A – by 0.001 in all observations where another individual (other than A) was already present. For observations where no other individual was present yet (and A was absent), we multiplied the probability of B’s presence by 0.999. After all of B’s sightings had been assigned, we chose a new individual from the 58 remaining and randomly selected an associate (i.e. for the second round either A or B) and followed the process as described above. We repeated this process until observations for all 60 individuals had been assigned. We then removed observations where no individual had been assigned, which resulted in 331 remaining observations (OR5).
